# Gut microbiome of migratory shorebirds: Current status and future perspectives

**DOI:** 10.1002/ece3.7390

**Published:** 2021-04-03

**Authors:** Zheng Zhang, Zhisong Yang, Lifeng Zhu

**Affiliations:** ^1^ Colleges of Life Sciences Nanjing Normal University Nanjing China; ^2^ Sichuan Academy of Giant Panda Chengdu China

**Keywords:** composition and function, energy requirement, migratory shorebirds, the gut microbiome

## Abstract

Migratory shorebirds have many unique life history characteristics, such as long‐distance travel between breeding sites, stopover sites, and wintering sites. The physiological challenges for migrant energy requirement and immunity may affect their gut microbiome community. Here, we reviewed the specific features (e.g., relatively high proportion of *Corynebacterium* and *Fusobacterium*) in the gut microbiome of 18 migratory shorebirds, and the factors (e.g., diet, migration, environment, and phylogeny) affecting the gut microbiome. We discussed possible future studies of the gut microbiome in migratory shorebirds, including the composition and function of the spatial‐temporal gut microbiome, and the potential contributions made by the gut microbiome to energy requirement during migration.

## Graphical abstract

We synthesised published 16S rRNA gene data in the migratory shorebirds (Cho & Lee, 2020; Grond et al., 2019, 2020; Risely et al., 2017, 2018), and found the predominant phyla of these 13 shorebird species include Firmicutes, Bacteroidetes, Proteobacteria, Fusobacteria, and Actinobacteria.

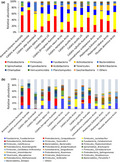

## 1 INTRODUCTION

Bird migration is the regular seasonal movement between breeding and wintering sites, and one common route is between north and south (Alerstam et al., 2003; Berthold, 2001). Out of these migratory birds, the shorebirds perform some of the longest and fasters migrations ever record (Gill et al., 2009). The shorebirds refer to species in the suborder (*Charadrii*) (such as plovers or sandpipers) that frequent seashore areas. Most shorebirds (e.g., *Calidris ruficollis*) travel long distances between breeding sites, stopover sites, and wintering sites (Gill et al., 2009). During long‐distance migration, they face specific physiological challenges, such as energy requirement, environmental pressure, and immunity (Buehler & Piersma, 2008; Klaassen, 1996; Weber, 2009). These extreme physiological challenges (e.g., body mass loss and partial atrophy of the gastrointestinal tract [McWilliams & Karasov, 2001; Piersma et al., 1999]) may affect the host–microbe interactions and lead the changes in the gut microbiome community (e.g., Cho & Lee, 2020; Grond et al., 2019; Risely et al., 2017, 2018).

The gut microbiome is defined as a combination of all the genomes of microorganisms in a specified environment (Grond et al., 2018). The gut microbiome plays an important role in the activities of the host (Kohl, 2012). Understanding the importance of the gut microbiome to bird host physiology and health is important, and studies have focused on the formation and maintenance of the gut microbiome and its effects on host fitness (Grond et al., 2018; Rooks et al., 2014; Waite & Taylor, 2015). Thus, the current studies on the gut microbiome of the migratory shorebirds mainly focus on the composition and specific microbes associated with the extreme physiological challenges during migration. Here, we reviewed the specific features of the gut microbiome of 18 migratory shorebirds (Table 1) and discuss possible future studies of the gut microbiome in migratory shorebirds.

**TABLE 1:** The information of the published gut microbiome data in 18 migratory shorebirds

Species	Sampling site	Location	Sampling time	Sample type	16s rRNA sequencing	References
*Himantopus himantopus*	Wintering grounds	Tagus Estuary, Portugal	2008–2009	Cloacal	16s full length, clone	Santos et al. (2012)
*Limosa limosa*	Wintering grounds	Tagus Estuary, Portugal	2008–2009	Cloacal	16s full length, clone	Santos et al. (2012)
*Tringa totanus*	Wintering grounds	Tagus Estuary, Portugal	2008–2009	Cloacal	16s full length, clone	Santos et al. (2012)
*Arenaria interpres*	Stopover site	Delaware Bay, USA	2011	Feces	V1‐V4, clone	Ryu et al. (2014)
*Calidris canutus*	Stopover site	Delaware Bay, USA	2011	Feces	V1‐V4, clone	Ryu et al. (2014)
*Calidris pusilla*	Stopover site	Delaware Bay, USA	2011	Feces	V1‐V4, clone	Ryu et al. (2014)
*Arenaria interpres*	Wintering grounds	Australia	2015–2016	Cloacal	V1‐V3, high throughput	Risely et al. (2017, 2018)
*Calidris acuminata*	Wintering grounds	Australia	2015–2016	Cloacal	V1‐V3, high throughput	Risely et al. (2017, 2018)
*Calidris ferruginea*	Wintering grounds	Australia	2015–2016	Cloacal	V1‐V3, high throughput	Risely et al. (2017, 2018)
*Calidris ruficollis*	Wintering grounds	Australia	2015–2016	Cloacal	V1‐V3, high throughput	Risely et al. (2017, 2018)
*Calidris alpina*	Breeding grounds	Arctic region	2011–2014	Feces	V3‐v4, high throughput	Grond et al. (2019)
*Calidris mauri*	Breeding grounds	Arctic region	2011–2014	Feces	V3‐v4, high throughput	Grond et al. (2019)
*Calidris melanotos*	Breeding grounds	Arctic region	2011–2014	Feces	V3‐v4, high throughput	Grond et al. (2019)
*Calidris pusilla*	Breeding grounds	Arctic region	2011–2014	Feces	V3‐v4, high throughput	Grond et al. (2019)
*Limnodromus scolopaceus*	Breeding grounds	Arctic region	2011–2014	Feces	V3‐v4, high throughput	Grond et al. (2019)
*Phalaropus fulicarius*	Breeding grounds	Arctic region	2011–2014	Feces	V3‐v4, high throughput	Grond et al. (2019)
*Phalaropus lobatus*	Breeding grounds	Arctic region	2011–2014	Feces	V3‐v4, high throughput	Grond et al. (2019)
*Pluvialis dominica*	Breeding grounds	Arctic region	2011–2014	Feces	V3‐v4, high throughput	Grond et al. (2019)
*Calidris alpina* US	Breeding grounds	Delaware Bay, USA	2018	Gut contents	V3‐v4, high throughput	Grond et al. (2019)
*Calidris pusilla* US	Breeding grounds	Delaware Bay, USA	2018	Gut contents	V3‐v4, high throughput	Grond et al. (2019)
*Calidris alba*	Breeding grounds	Northern Greenland	2017	Feces	V3‐v4, high throughput	Cho and Lee (2020)
*Actitis hypoleucos*	Unclear	Equatorial Guinea	2016	Gut contents	V3‐v4, high throughput	Capunitan et al. (2020)

## 2 *CORYNEBACTERIUM* ENRICHED IN THE GUT MICROBIOME OF THE RECENTLY ARRIVED MIGRATORY SHOREBIRDS POTENTIALLY RELATED TO THE ENERGY REQUIREMENT

**FIGURE 1:**
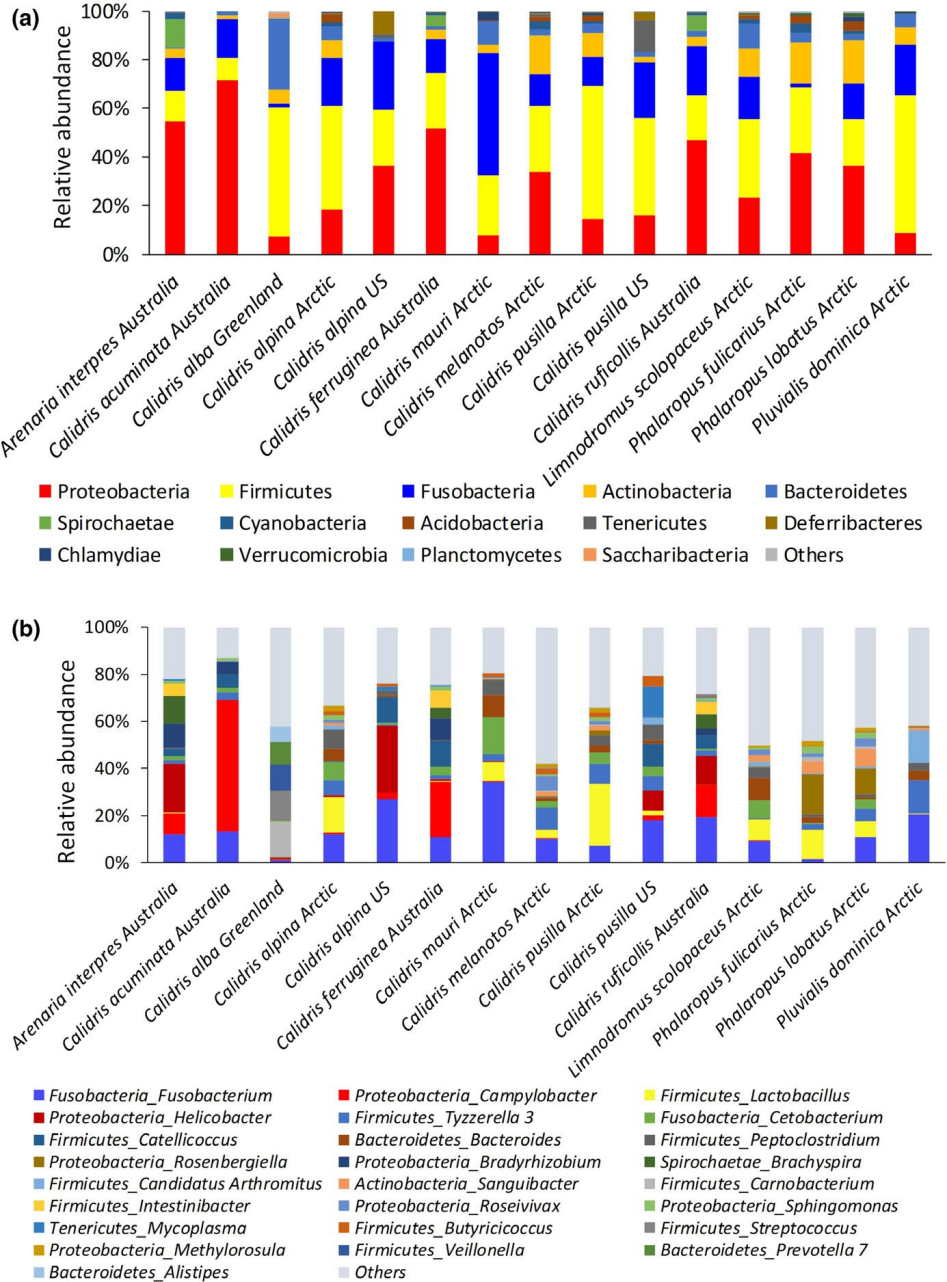
The analysis of the published bacterial 16S ribosomal RNA gene data (using high‐throughput sequencing) of the migratory shorebirds. (a) Phylum level. (b) Genus level. Details of the published data are shown in Table 1. The *trimmomatic* function was used for quality control on these raw datasets (Bolger et al., 2014). Operational taxonomic units (OTU) were defined as sharing >97% sequence identity by searching clean sequences against the SILVA132 database by pick‐up‐closed‐OTU methods (Christian et al., 2013). Taxon summary was created using the OTUs table (2,000 sequences per sample) in QIIME 1.9 (Caporaso et al., 2010)

We synthesized published 16S rRNA gene data (Table 1, 506 samples using high‐throughput sequencing technology) in the migratory shorebirds (Cho & Lee, 2020; Grond et al., 2019, 2020; Risely et al., 2017, 2018) and found the predominant phyla of these 13 shorebird species include Firmicutes, Bacteroidetes, Proteobacteria, Fusobacteria, and Actinobacteria (Figure 1). Firmicutes is also a dominant phylum in the other seven migratory shorebirds (Table 1): Clone sequencing: *Himantopus himantopus*, *Limosa limosa*, and *Tringa totanus* (Santos et al., 2012); clone sequencing: *Arenaria interpres*, *Calidris canutus*, and *Calidris pusilla* (Ryu et al., 2014); one sample: *Actitis hypoleucos* (Capunitan et al., 2020). Risely et al. (2017), Risely et al. (2018) revealed that the recent *Calidris* migrants (e.g., *Calidris ruficollis*) harbor a relatively higher proportion of *Corynebacterium* in the gut microbiome than that of the residents (Risely et al., 2017, 2018). *Corynebacterium*, one type of Gram‐negative bacteria, is a potential pathogen group in the human and animal gut (Bernard, 2012; Khamis et al., 2004), and the increased abundance of this genus may reflect the host immune response (Dorella et al., 2006; Rooks et al., 2014). The bacterial endotoxins (produced by some Gram‐negative bacteria) or exotoxins can lead to host inflammatory responses that increase fat deposition and energy harvest (Boulangé et al., 2016; Cani et al., 2009; Zhao, 2013). Thus, a high proportion of *Corynebacterium* in the recently arrived migrants may enable migrating shorebirds to maximize fat deposition (Risely et al., 2017, 2018). This specific feature in the migrant gut microbiome may also be associated with the physiological changes during the migration or an intestinal immune response (Risely et al., 2017, 2018). However, the strain level and real function of *Corynebacterium* in the migratory shorebird gut still not know and need to be investigated in the experiments (e.g., germ‐free animal models).

**FIGURE 2:**
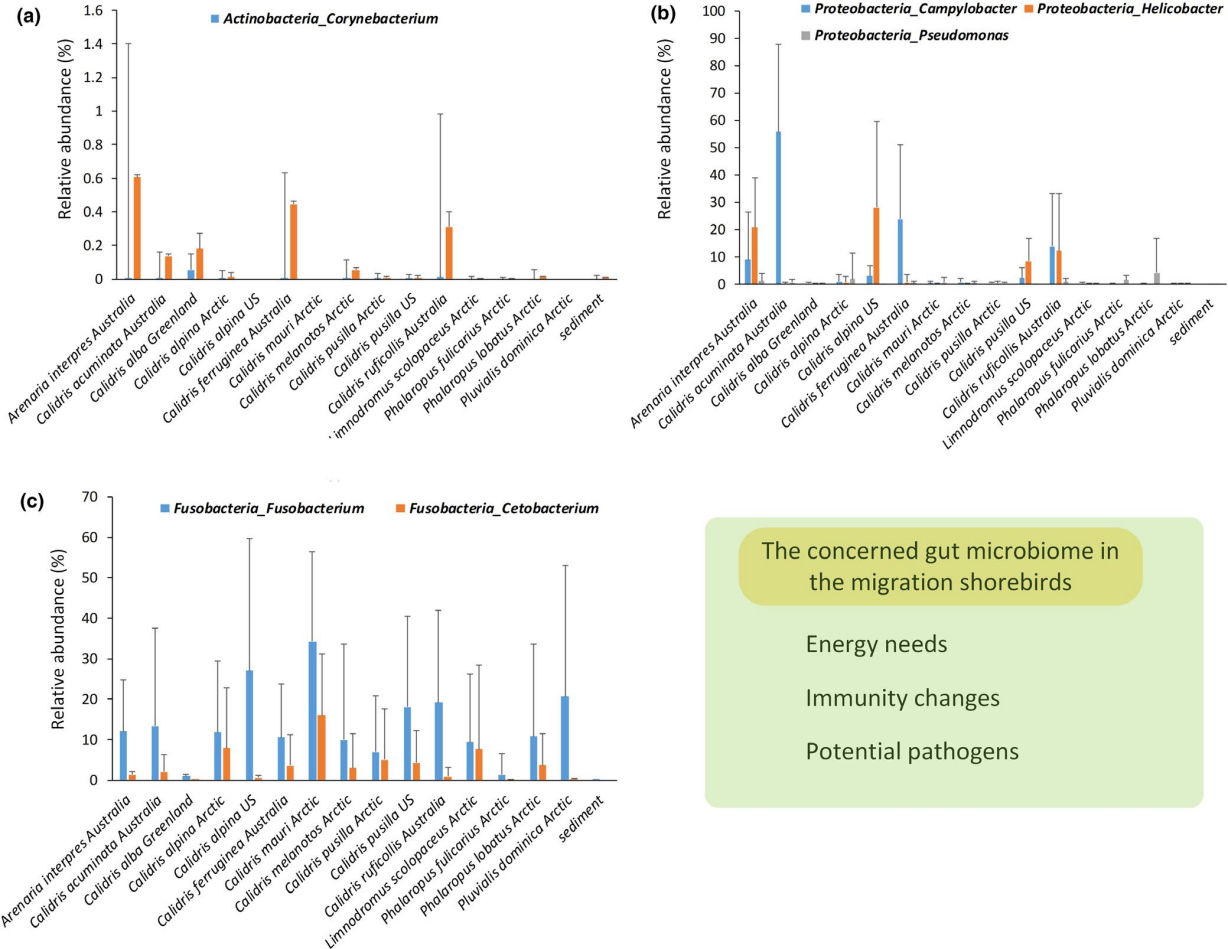
The specific features in the gut microbiome of the migratory shorebirds based on the analysis of the published bacterial 16S Ribosomal RNA gene data (using high‐throughput sequencing). (a) *Corynebacterium*. (b) *Fusobacterium* and *Cetobacterium* (c) *Campylobacter* and *Helicobacter*. Details of the published data are shown in Table 1

Moreover, the analysis of the published 16S rRNA gene data of the migratory shorebirds showed *Corynebacterium* was mainly enriched in *Calidris* shorebirds in Australia (Figure 2a). The abundance of *Corynebacterium* in the *Calidris* shorebirds in other regions (e.g., Arctic, Greenland, and US regions) was low (Figure 2a). Considering *Corynebacterium* was rare in the living environment (e.g., sediment [Risely et al., 2017]), we speculated that the different physiological stages (e.g., recently arrived migrants vs. breeding stage) might explain this difference in the abundance of *Corynebacterium* among these geographic populations. However, the difference in the dietary composition and living environment would also affect the migrant gut microbiome composition and function. Thus, we still need to investigate that whether this finding was the common pattern in the recently arrived migratory shorebirds along with different geographic populations.

## 3 A HIGH PROPORTION OF *FUSOBACTERIUM* IN THE GUT MICROBIOME OF THE MIGRATORY SHOREBIRDS POTENTIALLY RELATED TO THE CARNIVOROUS DIET

A high proportion of Fusobacteria (e.g., *Fusobacterium*) was identified in the gut microbiome of the migratory shorebirds in the Arctic region (Grond et al., 2019). The analysis on the published 16S rRNA gene data of the migratory shorebirds revealed another specific feature that most of the migratory shorebirds across different geographic populations (e.g., Australia, US, and Arctic regions) harbored a high proportion of Fusobacteria (mean abundance about 20 percent) (Figure 2b). Fusobacteria is the Gram‐negative and non‐spore‐forming anaerobes and may produce endotoxins (Tan et al., 1996; Warner et al., 1975). *Fusobacterium* is the common members of the gut microbiome in birds (Hird et al., 2015; Waite & Taylor, 2015), especially in the vulture (mean abundance about 10 percent) (Roggenbuck et al., 2014). A high proportion of *Fusobacterium* in the shorebirds' gut microbiome may result from the diet‐associated microbiome (e.g., biofilm comprised of microphytobenthos and bacteria) (Grond et al., 2019). Interestingly, a high proportion of *Fusobacterium* of the gut microbiome is also found in the alligators (mean abundance over 50 percent) (Keenan et al., 2013) and the meat‐eat carnivorans (mean abundance about 30 percent) (Zhu et al., 2018). For example, the high proportion of *Fusobacterium* in the vulture gut microbiome may indicate that vultures can benefit from the bacterial breakdown of carrion while tolerating bacterial toxins (Roggenbuck et al., 2014). Therefore, the common feature of the enriched Fusobacteria in the gut microbiome of these vertebrates might partially be related to their carnivorous or carrion diet.

## 4 A HIGH PROPORTION OF OPPORTUNISTIC PATHOGENS IN THE GUT MICROBIOME OF THE MIGRATORY SHOREBIRDS

Several bacteria, considered to be opportunistic pathogens, can cause bird morbidity and mortality (Kohl, 2012). The potentially pathogenic bacteria include Gram‐negative *Campylobacter* and *Helicobacter* in the migratory shorebird species (e.g., *Calidris* species) (Grond et al., 2020; Risely et al., 2018; Ryu et al., 2014; Santos et al., 2012). The analysis uncovered that several migratory shorebird's gut microbiome harbors a high proportion of *Campylobacter* and *Helicobacter* among geographic populations (Australia and US regions) (Figure 2c). The mean abundance of migratory *Calidris acuminate* in Australia was over 50 percent. As mentioned by Ryu et al. (2014), the migratory shorebirds are the important reservoirs of these opportunistic pathogens. *Campylobacter* lives in the digestive tract of animals such as wild birds, pigs, rodents, cats, and dogs (Acheson & Allos, 2001; Kaakoush et al., 2015; Moore et al., 2005). A high proportion of *Campylobacter* might reflect the high immune pressure in these migratory shorebirds under the physiological changes or the different environment during migration. However, the negative effect on the health in the migratory shorebirds is still unclear. In addition, given that specially enriched Gram‐negative bacteria (e.g., *Corynebacterium*, *Fusobacterium*, and *Campylobacter*) and their contradictory (pathogenic potential and energy harvest), the investigation on the function of migratory shorebirds' gut microbiome should focus on the interaction between the microbes and the host–microbe interaction.

## 5 HOST PHYLOGENY AND POTENTIAL SOCIAL CONTACT AFFECTING THE GUT MICROBIOME COMMUNITY OF THE MIGRATORY SHOREBIRDS DURING SYMPATRIC CLUSTERING

**FIGURE 3:**
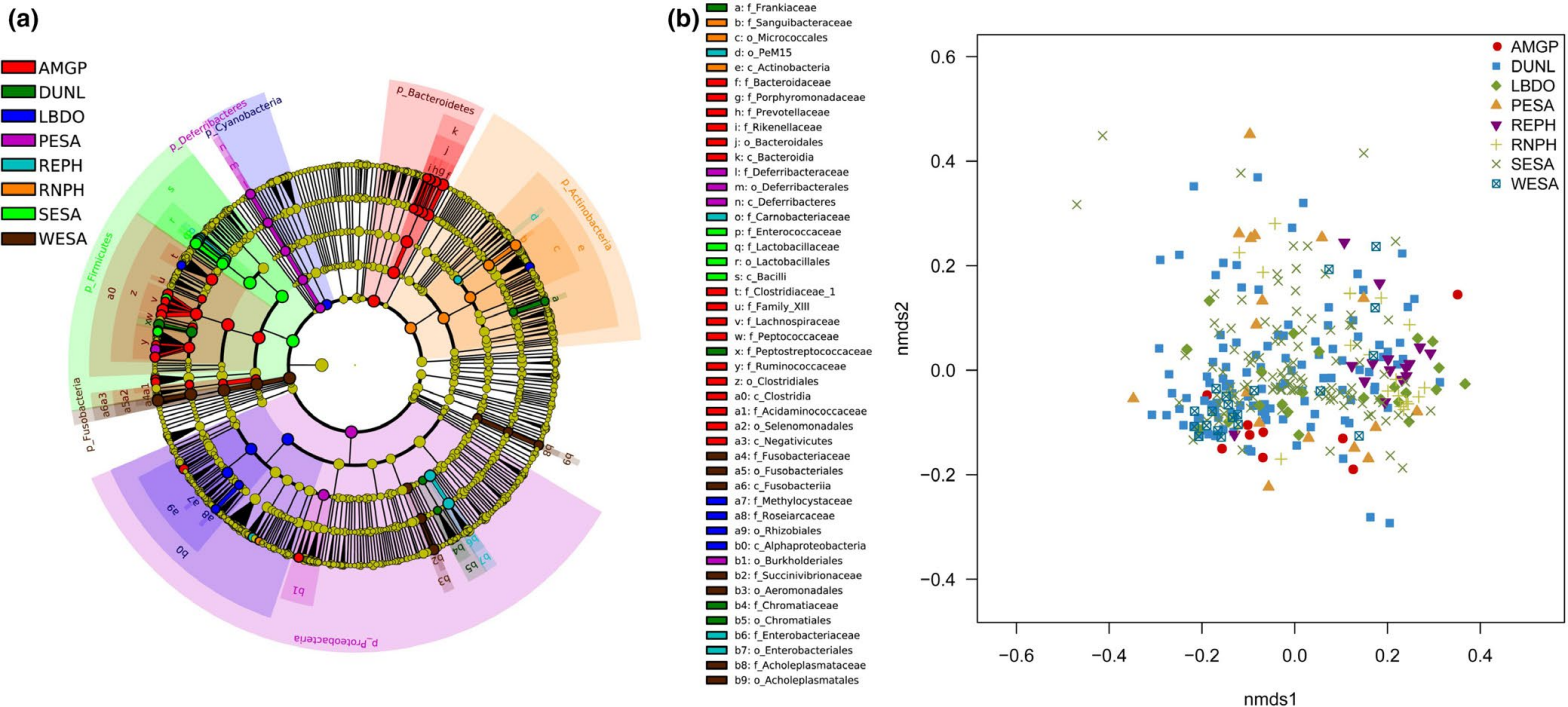
The re‐analysis on the gut microbiome community of eight migratory shorebirds (Grond et al., 2019). (a) Nonmetric multidimensional scaling (NMDS) using unweighted UniFrac distance displayed the mixed pattern in the gut microbiome among eight migratory shorebirds. (b) LEfSe (linear discriminant analysis effective size) determined the significant difference in the abundance of the gut microbial taxon among eight migratory shorebirds. AMGP, *Pluvialis dominica*; DUNL, *Calidris alpine*; LBDO, *Limnodromus scolopaceus*; PESA, *Calidris melanotos*; REPH, *Phalaropus fulicarius*; RNPH, *Phalaropus lobatus*; SESA, *Calidris pusilla*; WESA, *Calidris mauri*. The raw dataset of sediment microbiome came from Risely et al. (2017)

Most of the current research of the bird gut microbiome focus on nonmigratory birds, and in these species, host system development is the main driving force for the diversity of intestinal microbes (Grond et al., 2018; Waite & Taylor, 2015). The microbiome is a trait of the host species, and the evolutionary relationship between them and the potential shorebird phylogeny is assessed by comparative phylogenetic methods (Capunitan et al., 2020). The gut microbiome community may differ among different species of migratory shorebirds. Here, we re‐analyzed the raw dataset of eight migratory shorebird species in the Arctic region (Grond et al., 2019) and found a significant difference in the abundance of some gut microbiome among these species (Figure 3a). For example, Actinobacteria was enriched in *Phalaropus lobatus*, and Proteobacteria was enriched in *Calidris melanotos*. However, NDMS analysis using unweighted UniFrac distance displayed the mixed pattern in the gut microbiome community among these eight species (Figure 3b). These findings indicated the partial or weak effect on the gut microbiome by host phylogeny, and other factors might also affect the gut microbiome community.

The impact of shared habitat on the microbiota may be manifested in the sympatric clusters of migratory birds (Lewis et al., 2017). Social contact among intraspecies and interspecies may lead to the gut microbiome transmission and affect the gut bacterial communities in individual animals (Antwis et al., 2018; Archie & Tung, 2015; Grond et al., 2014; Kwong & Moran, 2016; Ryu et al., 2014). Different species of migratory shorebirds have direct or indirect contact because they share habitat and food during their migrations or breeding (Cho & Lee, 2020; Grond et al., 2019; Risely et al., 2017, 2018; Ryu et al., 2014; Santos et al., 2012). Therefore, we speculated that the effects of social contact on the gut microbiome community of the migratory shorebirds might also modify the effects contributed by host phylogeny.

## 6 THE POTENTIAL EFFECT BY ENVIRONMENT MICROBIOME

Risely et al. (2017) found red‐necked stints (*Calidris ruficollis*) acquired little environmental microbiome (about 0.1% of gut microbiome putatively from foraging sediment) (Risely et al., 2017). This indicated that these migratory shorebirds did not acquire microbes from the sediment during migration (Risely et al., 2017). This migratory shorebird species may resist the invasion of environmental microorganisms, which allows them to maintain a stable gut microbiome during migration from the breeding area to wintering sites (Risely et al., 2017). This study raised an interesting question on the gut microbiome transmission between the living environment (e.g., food source and living habitat source) in the migratory shorebirds during migration. The natural environment of the breeding site of some wild migratory shorebirds (such as the western sandpiper [*Calidris mauri*] and semipalmated sandpiper [*Calidris pusilla*]) can have a greater impact on the composition of intestinal microbes than phylogeny (Grond et al., 2019). The migratory shorebirds, during migration, would face different environments (e.g., winter sites, stopover sites, and breeding sites) (Gill et al., 2009). Currently, only one pilot study has tried to evaluate the contribution of the sediment‐source microbiome to the gut microbiome of *Calidris ruficollis* in winter sites (Risely et al., 2017). Thus, the relationship between the potential dynamics of migratory shorebirds' gut microbiome during long‐distance migration and the environment‐source microbial contribution is still unknown.

## 7 CONCLUSION

Here, we reviewed that the current studies mainly reveal the community in the migratory shorebird gut microbiome, which was potentially associated with the physiological challenges and energy requirement during migration. Thus, the next main scientific question related to common patterns and the function of the gut microbiome will be important and necessary.

## 8 FUTURE PERSPECTIVES

### 8.1 Spatial–temporal patterns of migratory bird gut microbiomes

**FIGURE 4:**
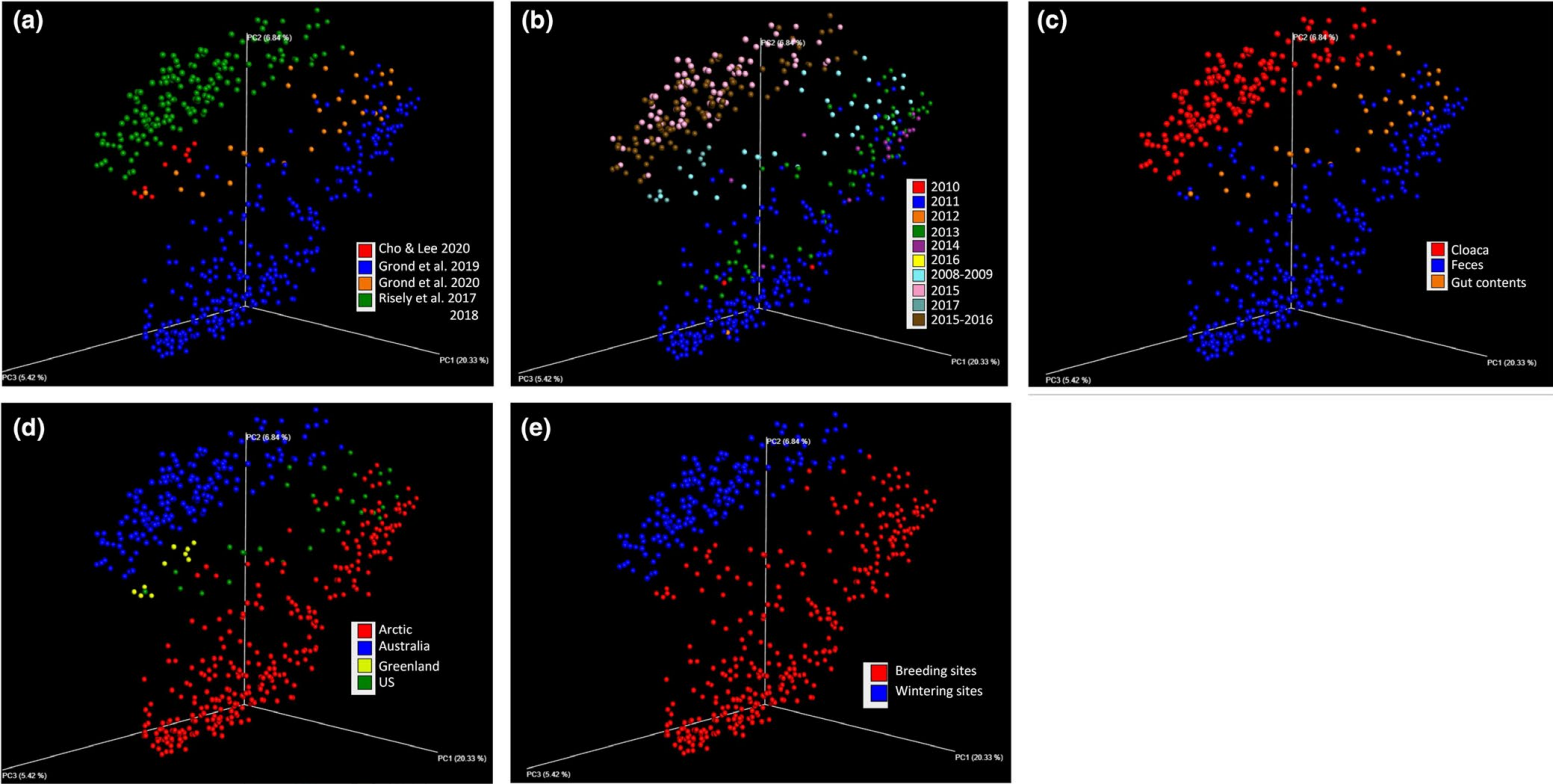
The PCoA analysis of the published bacterial 16S ribosomal RNA gene data of the migratory shorebirds using unweighted UniFrac distance. (a) The data source; (b) sampling time; (c) sample type; (d) study area; (e) sampling site. Details of the published data are shown in Table 1

**FIGURE 5:**
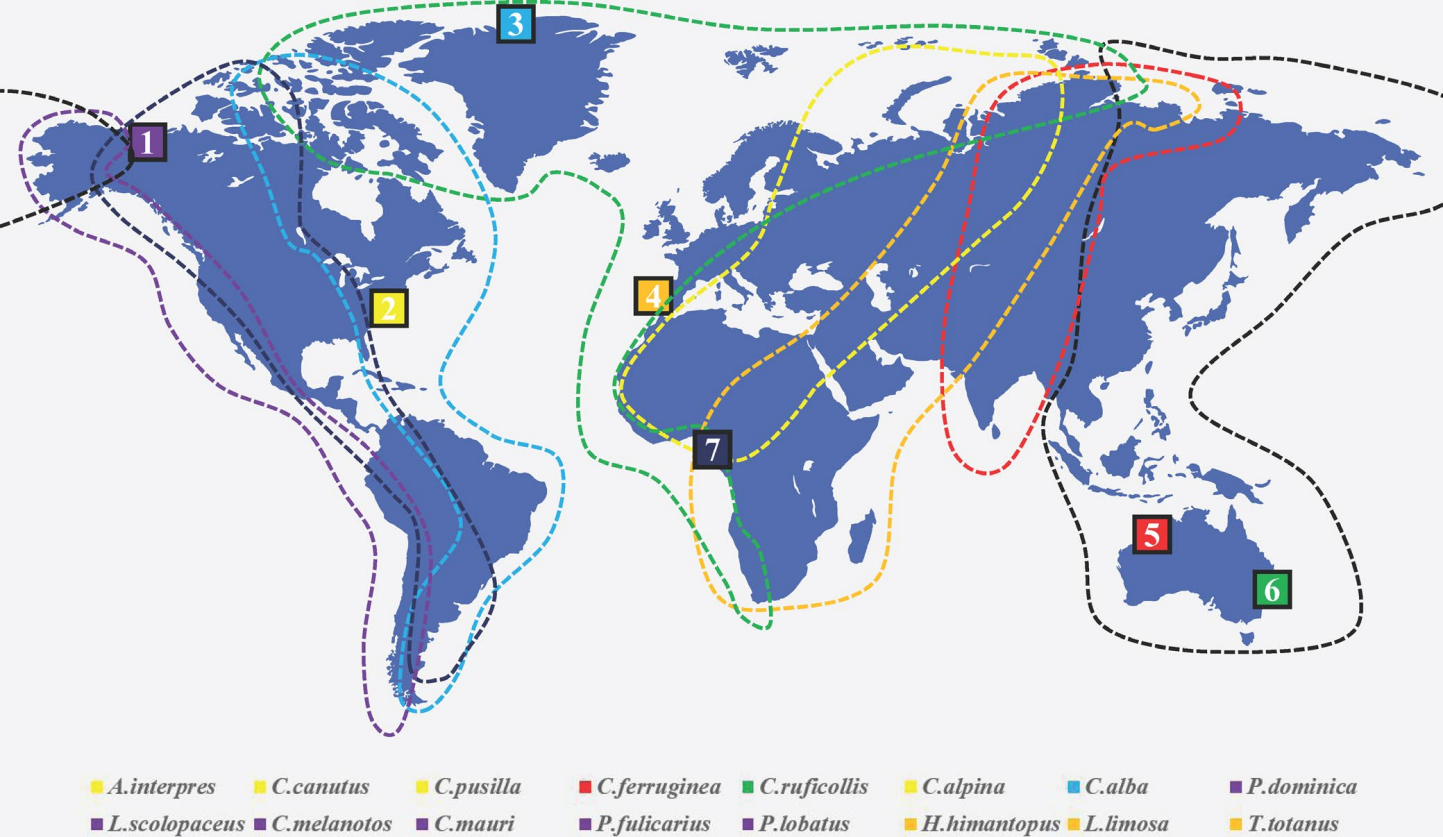
The main migration route worldwide (The sample location data were referred to published data: Cho & Lee, 2020; Grond et al., 2020; Grond et al., 2019; Risely et al., 2018; Risely et al., 2017; Ryu et al., 2014; Santos et al., 2012. The number represents the research location: 1 = MacKenzie River Delta in Alaska and Canada; 2 = Delaware Bay, USA; 3 = Sirius Passet; North Greenland; 4 = Tagus estuary, Portugal; 5 = Broome, Western Australia; 6 = Victoria, Eastern Australia. The migration route is redrawn according to the content of the URL https://www.sovon.nl/ and https://www.wwt.org.uk/. The black dotted line indicates the East Asia–Australia migration route. The red dotted line indicates the Central Asia–India migration route. The orange dotted line indicates the migration route from West Asia to East Africa. The yellow dotted line indicates the Mediterranean–Black Sea migration route. The green dotted line indicates the migration route in the eastern Atlantic Ocean. The cyan dotted line indicates the migration route of the western Atlantic Ocean. The blue dotted line indicates the Mississippi migration route. The purple dotted line indicates the migration route in the eastern Pacific Ocean)

Currently, it is hard to conclude which factors were mainly lead to the difference in the gut microbiome community of the migratory shorebirds during migration due to the differences in sampling time (not in the same migration season), geographic regions (not in the sample migration route), and sample types (Figure 4). The global distribution of sampling sites for migratory shorebirds research shows that data from Asia, Africa, and South America need to be supplemented (Figure 5). Therefore, to complete the global shorebirds data, these regions are key for future research, especially the migration route from Siberia to the eastern coast of China, which is significant for studying the community dynamics of the gut microbiome of migratory shorebirds. A large project would involve tracking changes in the composition of migratory shorebird gut microbiome and their living environment microbiome during migration, including premigration, migration, and end of the migration. This will be an international project, and it will face many challenges. The study will provide information on the relationship between the changes in the specific gut microbiota and migration. Studying the spatial–temporal changes of the gut microbiome during migration is significant for understanding the response of the dramatic physiological challenges and the survival status of migratory shorebird populations.

### 8.2 The function of the gut microbiome in migratory shorebirds (e.g., energy requirements)

The function of the gut microbiome is particularly important for migratory shorebirds, as they face extraordinary metabolic, nutritional, and immune challenges during migration (Altizer et al., 2011; Grond et al., 2019; Risely et al., 2018; Wikelski et al., 2003). Before migration, dunlin and semipalmated sandpipers will quickly gain weight and deposit fat (Grond et al., 2020). The trade‐off between microbial colonization and migratory bird energy requirement remains to be explored. Moreover, the function of gut microbiome (e.g., significant changes in some microbial group during migration) in the migratory shorebird gut needs to be deeply investigated in the experiments (e.g., germ‐free animals).

## CONFLICT OF INTEREST

The authors declared no conflicts of interest relevant to this manuscript.

## AUTHOR CONTRIBUTIONS

**Zheng Zhang:** Writing‐original draft (equal). **Zhisong Yang:** Writing‐original draft (equal). **Lifeng Zhu:** Conceptualization (equal); Formal analysis (equal); Funding acquisition (equal); Writing‐original draft (equal).

## Data Availability

Not applicable.
